# Surface Characteristics of Subtractively and Additively Manufactured Restorative Materials for Definitive Restorations

**DOI:** 10.3390/ma18184222

**Published:** 2025-09-09

**Authors:** Konstantinos Tzimas, Maria Dimitriadi, Christos Rahiotis, Eftychia Pappa

**Affiliations:** 1 Department of Operative Dentistry, School of Dentistry, National and Kapodistrian University of Athens, 11527 Athens, Greece; kwstastzimas@dent.uoa.gr (K.T.); effiepappa@dent.uoa.gr (E.P.); 2 Department of Biomaterials, School of Dentistry, National and Kapodistrian University of Athens, 11527 Athens, Greece; mardimit@dent.uoa.gr

**Keywords:** CAD-CAM, surface properties, wettability, FTIR, composite resins, biomaterials

## Abstract

Advancements in Computer-Aided Design/Computer-Aided Manufacturing (CAD/CAM) have promoted the development of novel dental materials for several types of definitive restorations. The aim of this study was to evaluate the surface characteristics of resin-based CAD/CAM restorative materials, fabricated using both subtractive and additive manufacturing techniques. The materials tested included Filtek Z550 (FZ), Vita Enamic (VE), Shofu HC (SH), and VarseoSmile TriniQ (TQ). For each material, 24 specimens were prepared; half were manually polished following the manufacturer’s recommendations, while the other half underwent standardized metallographic polishing. The surface roughness, wettability, and molecular composition were assessed. A statistical analysis was performed using IBM SPSS 29.0 at a 95% confidence level (α = 0.05). Statistically significant differences in surface properties were observed between direct and indirect restoratives following manual polishing, with SH performing favorably in terms of surface roughness. The polishing method significantly influenced the surface characteristics of each material, except for certain roughness parameters in SH. Both surface roughness and wettability were dependent on the material and the polishing technique, highlighting the need for improved material-specific polishing protocols.

## 1. Introduction

In recent decades novel classes of dental restorative materials have been developed, driven by advances in filler technology, coupling agents, and polymer chemistry. These innovations have enhanced the mechanical properties, polishability, and longevity of resin-based materials [1,2,3]. In parallel, the advent of digital dentistry has further facilitated the development of CAD/CAM restorative materials manufactured through both subtractive (the milling of solid blocks or disks) and additive (layer-by-layer fabrication) techniques [4,5].

CAD/CAM restorative materials for definitive restorations can be broadly categorized into ceramic and resin-based materials [6]. Resin-based CAD/CAM restorative materials are increasingly used in clinical applications due to their enhanced machinability, ease of intraoral repair, and mechanical properties that are adequately suited for clinical use [7,8]. Moreover, their physical characteristics, such as their modulus of elasticity, which closely resembles that of natural tooth structures, enhance their clinical performance [9,10]. Finally, occlusal and proximal adjustments are easily accomplished [11]. The implementation of the hybrid ceramic CAD/CAM restorative material Vita Enamic (Vita Zahnfabrik) marked a significant innovation in resin-based dental material science. It is classified as a polymer-infiltrated ceramic network (PICN) material, with a dual-structure design [12]. The porous, pre-sintered ceramic network infiltrated with a polymer phase synergistically combines ceramic strength with polymer resilience, mimicking natural tooth properties, while providing long-term durability and esthetics [13,14]. Similarly, numerous milled resin-based CAD/CAM materials reinforced with dispersed fillers are formulated with optimized filler loading and matrix design to improve mechanical performance, reduce brittleness, and enhance machinability [8,9,12,15]. Simultaneously, the three-dimensional printing (3D printing) of composite resins has expanded the potential of restorative materials. The additively manufactured resin-based restorative materials—primarily fabricated by vat polymerization techniques, such as stereolithography (SLA), digital light processing (DLP), and liquid crystal display (LCD) printing [16,17]—enable chairside fabrication, reduce material waste and production cost, and allow the precise reproduction of complex geometries compared with their milled counterparts [16,18]. The lack of scientific evidence on 3D-printed resin-based materials for definitive restorative applications remains evident [19]. While additive manufacturing provides satisfactory esthetics, adequate fit, time efficiency, and cost-effectiveness [20,21], the high concentration of organic compounds required to maintain the material’s liquid consistency [22] may compromise its mechanical and clinical performance [19,23].

Although the mechanical behavior of milled and 3D-printed restorative materials for definitive restorations has been extensively studied, research addressing their surface properties remains comparatively limited. Several studies have examined the surface characteristics of subtractively manufactured restorative materials, particularly in relation to biofilm formation. Their divergent results underscore the need for further investigation [24]. In contrast, the surface properties of additively manufactured restorative materials remain largely unexplored, indicating a significant gap in the current literature.

In addition to restorative materials, dental manufacturers have developed specially designed polishing systems to achieve a high-gloss appearance on the final restoration. An important consideration is the extent to which both material type and polishing procedures influence surface characteristics—such as roughness and wettability—as these properties are inextricably linked to plaque accumulation and secondary caries [25,26,27,28]. Furthermore, surface roughness is associated with wear resistance, color stability, aesthetics, and the overall longevity of the restoration [29,30].

In response to the limitations of current research, this in vitro study aimed to evaluate the surface characteristics of novel materials representing distinct categories of contemporary CAD/CAM restorative options, after polishing with manufacturer-recommended protocols, and to compare these surfaces with those achieved through optimal metallographic polishing. The comparison sought to determine the extent to which clinical polishing protocols approximate the optimal polishing capacity of each material. Therefore, two null hypotheses were formed:

**H_0_** **1.** *There are no statistically significant differences in the surface properties among the tested materials when subjected to either the clinically recommended or the optimized metallographic polishing procedures.*

**H_0_** **2.** *There are no statistically significant differences in surface characteristics between the clinically recommended polishing procedures and the corresponding optimized metallographic procedures for each material.*

## 2. Materials and Methods

### 2.1. Material Selection

Four different restorative materials with distinct compositions, representing four categories of contemporary restorative modalities, were included in this study. All selected materials are indicated for single, extensive, definitive restorations. A nanohybrid composite resin was used as the control, as direct composite resins remain clinically relevant for comparison due to their long-standing use as a benchmark in restorative dentistry. Details on the material compositions are presented in Table 1.

### 2.2. Sample Size Calculation

A sample size calculation was performed using the G*Power 3.1 software program (G*Power, Heinrich-Heine Universität, Düsseldorf, Germany). The analysis was conducted with an alpha level of 0.05, a statistical power of 0.90 (90%), and a medium effect size for each tested parameter (material, polishing procedure, and their interaction), as determined by a pilot study. The smallest medium effect size was observed for the parameter *Sds* (f = 0.34), which was used as the basis for the calculation. Therefore, the total sample size was calculated at 93, taking into account the evaluation of 4 materials and 2 polishing procedures (a total of 8 groups). This corresponds to 24 samples per material group and 12 samples per polishing procedure within each material group.

### 2.3. Specimen Preparation

A total of ninety-six (96) specimens with standardized dimensions of 10 mm (width) × 12 mm (length) × 2 mm (height) were fabricated for each material. The direct composite resin specimens were fabricated by incrementally placing the material into transparent thermoplastic molds, which were created from a 3D-printed model with specified dimensions. These molds were pressed between glass plates covered with a Mylar strip and then light-cured for 20 s using an LED curing unit (Elipar Deep Cure—L LED curing light, with an intensity of 1.47 mW/cm^2^, 3M ESPE, St. Paul, MN, USA). Specimens of the milled CAD/CAM materials (SH and VE) were fabricated using a 0.3 mm thick diamond wheel embedded in a low-speed precision cutting machine under constant water cooling. For the 3D-printed material, a model with the specified dimensions was designed using SolidWorks software (SOLIDWORKS 2022 version, Dassault Systèmes, Vélizy-Villacoublay, France) and exported as an STL file. The file was imported into a nesting software program (AsigaComposer 2.0, Alexandria, NSW, Australia) and positioned horizontally on the build platform, without supports. The layer thickness was set to 50 μm, and a DLP 3D printer was selected to fabricate the specimens (ASIGA MAX 405, Alexandria, NSW, Australia). Following the manufacturer’s recommendations, cleaning and post-curing protocols were employed [34]. The dimensions of all specimens were verified using a digital caliper (±0.01 mm).

### 2.4. Polishing Procedures

All specimens of each material (n = 24/material) were initially ground on a polishing unit (DapV, Struers, Copenhagen, Denmark) using silicon carbide (SiC) abrasive papers up to 1200 grit size to create a uniform baseline surface. Subsequently, half of the specimens from each material group (n = 12/material) were polished according to each manufacturer’s specified polishing protocol recommended for each restorative material. More precisely, the direct composite FK, was manually polished using a four-stage aluminum oxide disk system, without polishing paste [31]. The milled composite SH was polished with a four-step silicon carbide and aluminum oxide disk polishing system, followed by the application of two polishing pastes [32]. The milled hybrid ceramic material VE underwent manual polishing using a two-step polishing system, followed by the application of a diamond polishing paste [33]. Lastly, the 3D-printed composite was polished using a pumice stone and a universal polishing paste [34]. For each specimen, a new polishing disk, lens, or goat hair bristle was used to ensure consistency and prevent cross-contamination. The remaining specimens (n = 12/material) were subjected to standardized optimal metallographic grinding up to 4000 grit, followed by high-performance polishing using polishing cloths and diamond suspensions and lubricants. Following polishing, specimens were ultrasonically cleaned in distilled water at 37 °C for 10 min. Details of the polishing procedures performed are presented in Table 2.

### 2.5. Surface Roughness Evaluation

Surface roughness was analyzed using an optical interferometric profiler (Wyko NT 1100, Veeco, Tucson, AZ, USA). Measurements were performed under the following conditions: a 20× Mirau lens, a 2× field of view, an analysis area of 148 × 113 μm^2^ (41.6 × effective magnification), vertical scanning mode, 2% modulation, and tilt correction. For each specimen, three measurements were taken from the top surface, and the mean value was recorded as the representative result. Next, 3D profilometric images were acquired and the 3D surface roughness parameters evaluated were as follows: The amplitude parameters Sa (arithmetic mean of the absolute values of the surface height deviations measured from the best fitting plane) and Sz (average difference between the 5 highest peaks and 5 lowest valleys—10 height points over the complete 3D surface); the spatial parameter Sds (summit density, the number of summits per unit area making up the surface); the hybrid parameter Sdr (developed interfacial area ratio, the percentage of additional surface area contributed by the texture as compared to an ideal plane the size of the measurement region); and the functional parameters Sc (core void volume, the volume the surface would support from 10 to 80% of the bearing ratio) and Sv (surface void volume, the volume the surface would support from 80% to 100% of the bearing ratio) [35]. To support surface characterization, representative images of each material were obtained at 50× magnification using a reflection mode optical microscope (DM4000B, Leica Microsystems, Wetzlar, Germany).

### 2.6. Surface Wettability Assessment

Surface wettability was assessed by measuring the water contact angles via the sessile drop method [36]. A 2.0 μL droplet of ultrapure water was dispensed onto the specimen surface using a precision micro-pipette. The contact angle (θ) formed was measured 10 s after deposition by an optical goniometer (Ossila L2004A1, Leiden, The Netherlands). This 10 s interval was chosen to allow the droplet to stabilize while minimizing evaporation, which can reduce the measured contact angle [36]. The droplet profile was captured, and the left, right, and mean contact angles for each measurement were calculated. All measurements were performed under controlled environmental conditions (23 °C, 50% RH). For each specimen, three measurements were taken at distinct locations to ensure measurement accuracy and repeatability; the average value was then used for further analysis.

### 2.7. Attenuated Total Reflectance–Fourier Transformed Infrared Spectroscopy (ATR-FTIR) Analysis

One unpolished specimen of each material and one polished sample of each material, either manually or metallographically, were subjected to ATR-FTIR analysis to obtain qualitative information on the chemical changes induced in the material surfaces after polishing. A single-reflection ATR accessory (Golden Gate, Specac, Orpington, Kent, UK) with a diamond crystal (2 × 2 mm) and ZnSe lenses were attached to an FTIR spectrometer equipped with a deuterated l-alanine doped triglycine sulfate detector (Spectrum GX, Perkin-Elmer Corp, Buckinghamshire, Bacon, UK). Surfaces were pressed against the refractive crystal by a sapphire anvil to achieve firm contact with the diamond crystal, and spectra were recorded under the following conditions: a spectral range of 4000−650 cm^−1^, a resolution of 4 cm^−1^, and 20 scans coadded. The depth of analysis was estimated at 1.66 μm at 1000 cm^−1^.

**Figure 1:**
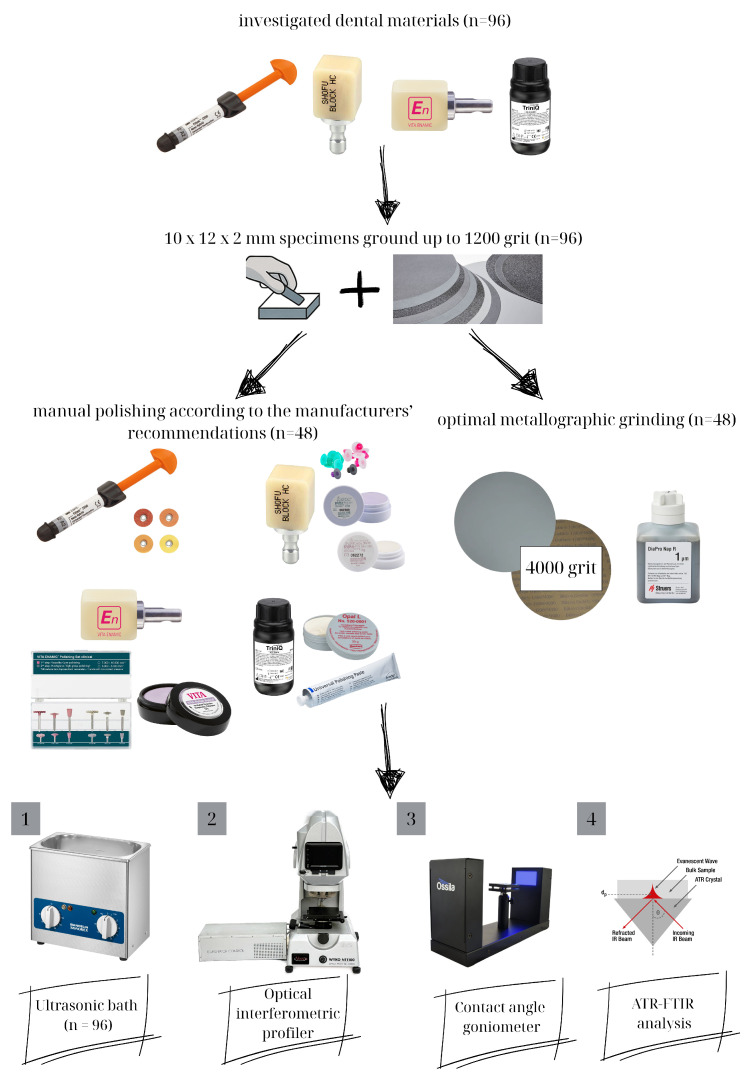
The study design flowchart.

The study design is described in detail in Figure 1.

### 2.8. Statistical Analysis

The assumption of normality was evaluated using the Shapiro–Wilk test, and the homogeneity of variances was assessed using Levene’s test. Based on the results of these preliminary tests, one-way ANOVA and Welch’s robust test of equality of means were performed separately for each polishing procedure to compare the means among all four independent material groups. Post hoc pairwise comparisons were performed using the Bonferroni correction when equal variances were assumed, and the Games–Howell correction when variances were unequal, to identify statistically significant differences between groups. A multiple linear regression analysis was conducted to determine which roughness parameters significantly affected wettability. An independent samples *t*-test was performed to assess the differences in surface characteristics between metallographically polished and manually polished specimens within the same material group (Student’s *t*-test when equal variances were assumed and Welch’s *t*-test when a violation of the equality of variances occurred). A generalized linear model (GLM) was applied to the surface characteristics. Each surface parameter was analyzed separately as a dependent variable. The model included the main effects of material and polishing procedures, as well as their interaction, treated as fixed factors. Since the data followed a normal distribution but violated the assumption of the homogeneity of variances, a normal distribution with an identity link function was used. Statistical significance was set at *a* = 0.05. Statistical analysis was performed using IBM SPSS Statistics software version 29.0 (IBM Corp, Armonk, New York, NY, USA).

## 3. Results

### 3.1. Surface Roughness Evaluation

**Figure 2:**
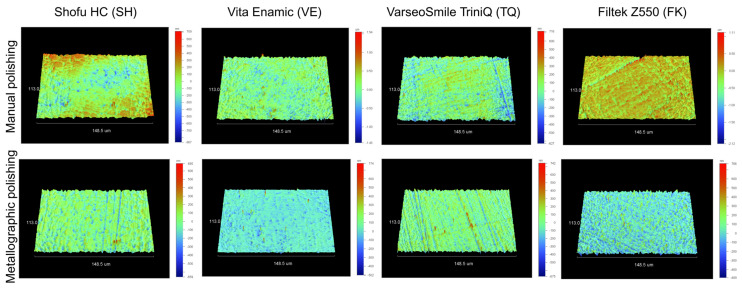
Representative 3D optical profilometric images of each material, comparing surface topographies obtained after manual and metallographic polishing (148.5 × 113 μm scan size, amplitude range for manual polishing: SH: 1.58 μm, VE: 2.97 μm, TQ: 1.34 μm, FK: 3.23 μm; amplitude range for metallographic polishing: SH: 1.32 μm, VE: 1.28 μm, TQ: 1.42 μm, FK: 1.26 μm).

**Figure 3:**
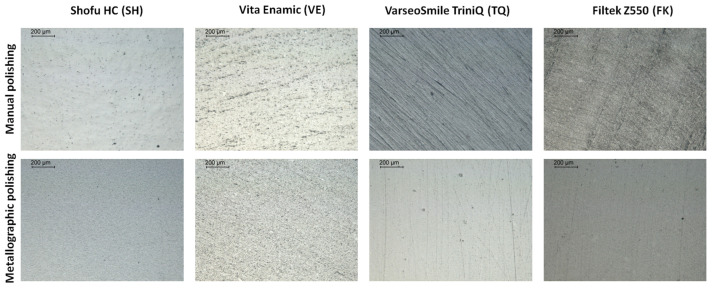
Representative optical microscope images of manually and metallographically polished specimens for each material group (50× magnification, bar: 200 μm).

Representative 3D profilometric images and optical microscope images (50× magnification, reflection mode) of the materials following the respective polishing procedures are presented in Figure 2 and Figure 3, respectively. Based on the 3D profilometric images, metallographic polishing yielded more uniform surface topographies for most materials, as indicated by a lower amplitude range. In the optical microscope images, the metallographically polished specimens exhibited more homogeneous, smooth, and scratch-free surfaces compared with those subjected to manual polishing. The latter displayed strong evidence of polishing tracks and surface defects, associated with the polishing methodology used for the majority of the materials.

Table 3 presents the detailed results for all evaluated surface roughness parameters.

Among the manually polished materials, statistically significant differences in surface roughness parameters were observed. The ranking of the statistically significant differences was as follows: FK > VE, TQ > SH for Sa, Sz, Sdr, and Sv; SH, VE, TQ > FK for Sds, and FK > VE, TQ > SH for Sc. Overall, the CAD/CAM materials outperformed the conventional composite resin, with SH exhibiting the most favorable surface roughness characteristics across all parameters, except for Sds.

All evaluated surface roughness parameters showed statistically significant differences among the metallographically polished materials. Specifically, for the amplitude parameter Sa, FK, SH, and TQ exhibited significantly higher mean values than VE, whereas TQ showed no significant difference from FK but differed significantly from SH. Regarding the amplitude parameter Sz, FK and VE demonstrated significantly greater values compared with SH. The mean Sz values of TQ were not significantly different from either FK or VE or from SH. The ranking of the statistically significant differences concerning Sdr was as follows: FK > SH, while VE and TQ showed no significant differences from either group. For Sds, the ranking was SH, TQ, HC > FK, suggesting that all CAD/CAM materials exhibited significantly higher mean Sds values compared with the control group. The statistically significant rankings for Sc are FK > TQ > VE and SH > VE, while SH showed no significant differences from both FK and TQ. Lastly, SH demonstrated the highest mean Sv values compared with both the direct and indirect restorative materials (SH > VE, FK, TQ). Among all materials, VE achieved the lowest values for Sa, Sdr, Sc, and Sv, indicating superior surface smoothness and reduced functional roughness. However, it exhibited relatively high values in Sz and Sds, reflecting a greater peak-to-valley height and spatial density.

Comparisons between manually and metallographically polished specimens were performed and statistically significant differences between the polishing procedures within each material group were revealed. Specifically, VE, TQ, and FK exhibited significantly higher mean values for the Sa, Sz, Sdr, Sc, and Sv surface parameters following manual polishing compared with optimal metallographic grinding. In contrast, SH did not exhibit significant differences between the two polishing methods concerning these parameters. Regarding Sds, insignificant differences were displayed for FK and VE, whereas SH and TQ exhibited significantly higher values following metallographic polishing.

**Figure 4:**
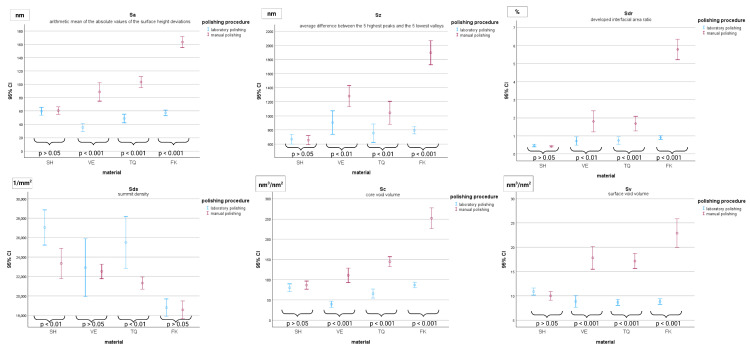
Comparison of surface characteristics between polishing procedures within each material group, including mean values, 95% confidence intervals, and the corresponding *p*-values for all comparisons.

Figure 4 illustrates the differences in the surface roughness parameters between manual and metallographic polishing within each material.

### 3.2. Surface Wettability

**Figure 5:**
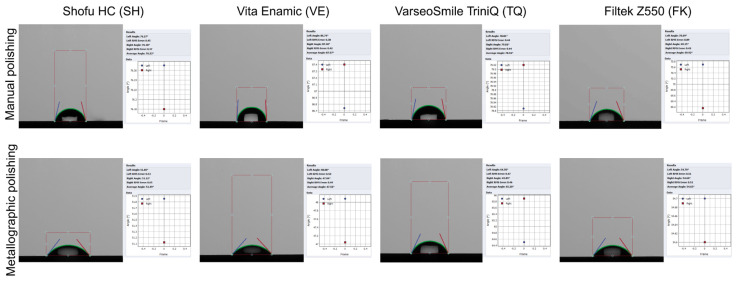
Representative wettability images of manually and metallographically polished specimens for each material group.

Representative wettability images of the tested surfaces are illustrated in Figure 5. The ranking of statistically significant differences after manual polishing was VE > TQ > SH > FK, whereas after metallographic polishing, the order changed to TQ > FK > SH > VE. All mean values between the two polishing conditions within each material differed significantly, with manually polished specimens exhibiting greater contact angle measurements. The CAD/CAM materials demonstrated significantly higher contact angles compared with the conventional composite resin following manual polishing. All in all, both polishing procedures presented contact angles below 90°, indicating a hydrophilic surface characteristic for all the tested materials. The detailed water contact angle results are shown in Table 4.

**Figure 6:**
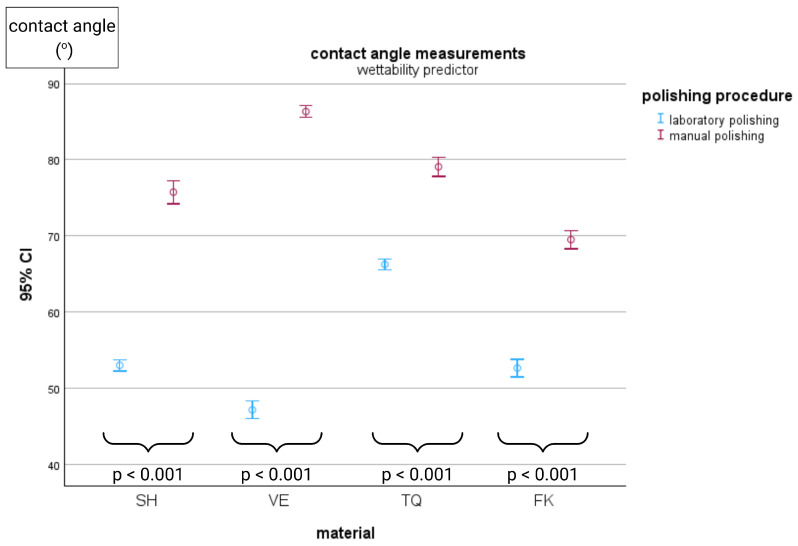
Comparison of surface characteristics between polishing procedures within each material group, including mean values, 95% confidence intervals, and the corresponding *p*-values for all comparisons.

Figure 6 illustrates the differences in contact angle measurements between manual and metallographic polishing within each material.

### 3.3. Interaction Between Surface Roughness Parameters and Surface Wettability

A multiple linear regression analysis was conducted to evaluate the influence of surface texture parameters on contact angle measurements for both manually polished and laboratory-polished materials. The model significantly predicts wettability (*F*(6, 89) = 13.799, *p* < 0.001), confirming that at least one of the examined surface roughness parameters contributes to explaining the variations in contact angle values. The model demonstrates a moderate effect size, accounting for 48.2% of the variance in wettability (R^2^ = 0.482, *p* < 0.001). Among the tested surface parameters, only Sdr was a statistically significant predictor (*B* = –9.99, *p* < 0.001), indicating that increased Sdr is associated with decreased contact angles and thereby greater hydrophilicity. More precisely, for every unit increase in Sdr the contact angle is decreased by almost 10^0^. However, due to intercorrelations among the roughness parameters, this estimate should be interpreted with caution.

### 3.4. Development of a Generalized Linear Model (GLM) to Assess the Effect of Material Type and Polishing Procedure on the Measured Surface Characteristics

The fundamental principle of the model is that material type significantly influences all surface characteristics. Additionally, the polishing procedure had a significant impact on surface characteristics, and the effect of polishing on these characteristics depended on the material used.

Based on the generalized linear model, which used the manually polished FK as the reference, all three CAD/CAM materials (SH, VE, and TQ) showed a significant decrease in the Sa, Sz, Sdr, Sc, and Sv surface roughness parameters relative to the reference group (*p* < 0.001). Metallographic polishing resulted in a significant decrease in the mean values of those parameters and the effect of polishing on these parameters depended on the material type (*p* < 0.001). The reduction effect of the metallographic polishing was mitigated depending on the material type with a greater moderation presented for the SH material (*p* < 0.001). Regarding the Sds parameter, SH, VE, and TQ showed a significant increase in Sds compared with the control group (*p* < 0.001). The increase in Sds after metallographic polishing was not statistically significant compared with manual polishing (*p* > 0.05). The effect of polishing on Sds depended on the material type for SH (*p* < 0.05) and TQ (*p* < 0.01), but not for VE (*p* > 0.05). Lastly, regarding water contact angles, all CAD/CAM materials (SH, VE, and TQ) exhibited a significant increase in contact angles relative to the reference group (*p* < 0.001). Metallographic polishing resulted in a significant decrease in contact angles (*p* < 0.001), and the effect of polishing on water contact angles depended on the material type (*p* < 0.001). A detailed table with the results of the GLM is provided as Table S1 in the Supplementary Materials Section.

### 3.5. ATR-FTIR Analysis

**Figure 7:**
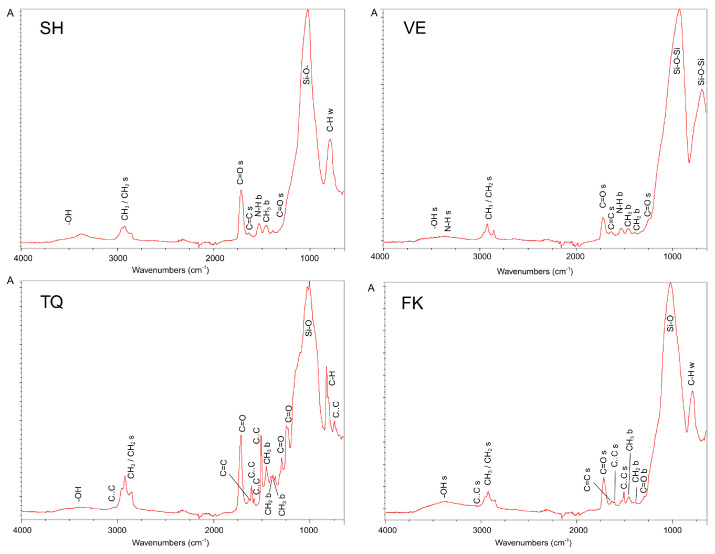
Absorbance ATR-FTIR spectra of reference materials with peak annotations.

The ATR-FTIR spectra of all investigated materials before manual and metallographic polishing (intact composite resin slabs, milled CAD/CAM blocks, and 3D-printed specimens) along with the corresponding characteristic peak assignments are illustrated in Figure 7. The spectra of VE and SH are free of aromatic compounds and are based on aliphatic urethane monomers, as observed by the presence of N–H peaks. VE demonstrated the lowest relative intensity of the organic phase peaks. In contrast, TQ and FK exhibited characteristic peaks attributed to aromatic vibrations.

**Figure 8:**
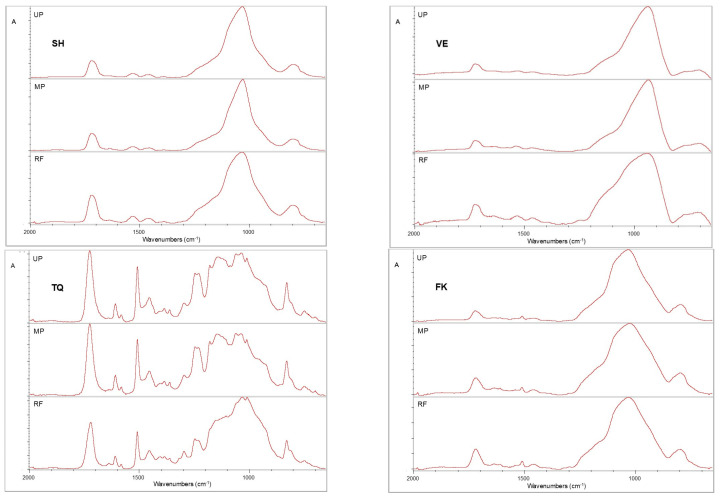
Expanded absorbance ATR-FTIR spectra of reference (RF), manually polished (MP), and metallographically polished (UP) materials demonstrating changes in the relative intensity of the ester peak (1725 cm^−^^1^) compared with the major complex peak around 1100 cm^−^^1^ mainly assigned to inorganic compounds.

Expanded spectra in the 2000–650 cm^−1^ range for reference, unpolished specimens as well as for both the manually and metallographically polished materials within each material group are depicted in Figure 8. A slight reduction in the relative intensity of the organic phase peaks (mainly the C=O peak at 1725–1707 cm^−1^) compared with the strong Si–O peak of the inorganic phase was observed in the SH (1020 cm^−1^) and VE (933 cm^−1^) specimens after polishing. A similar pattern was observed in FK, with metallographic polishing resulting in a significant reduction in the relative intensity of the organic C=O peak. In contrast, the polishing of TQ resulted in an increase in the C=O peak compared with the inorganic phase which demonstrated two well defined shoulders at a higher frequency. The differences in organic phase content were semi-quantitatively assessed by calculating the peak height ratios of C=O to Si–O for the manually polished (MP) and metallographically polished (UP) states. The obtained ratios were normalized to the corresponding values of each material and expressed as percentages. The results (means ± standard deviations from three randomly selected specimens) indicated a reduction in the organic phase in all materials after polishing [−47 ± 6% (MP) and −43 ± 4% (UP) for VE; −42 ± 7% (MP) and −41 ± 4 (UP) for SH; −26 ± 3% (MP) and −56 ± 8% (UP) for FK], with the exception of TQ, which exhibited an increase in the organic phase [72 ± 9% (MP) and 86 ± 7% (UP)].

## 4. Discussion

This in vitro study revealed statistically significant differences in surface roughness and surface wettability among all tested restorative materials, both across different materials and between the two polishing procedures. The results highlight the critical role of material type, polishing procedure, and their interaction on the surface characteristics. Therefore, the two null hypotheses should be rejected.

The four selected materials represent distinct categories of current restorative options: direct composite resins (FK), milled resin-based materials with dispersed fillers (SH), milled hybrid ceramics (VE), and 3D-printed resin-based materials (TQ). The inclusion of a conventional composite resin as the control group corresponds to a logical and clinically relevant choice, given its established role as a reference standard in restorative dentistry. This aligns with previous in vitro studies assessing the surface characteristics of novel resin-based CAD/CAM materials [37,38,39,40]. Including representatives from each material category enables a comprehensive evaluation of surface characteristics across subtractive and additive manufacturing technologies, reflecting modern developments in digital dentistry and materials science.

To the best of our knowledge, this in vitro study is among the first to directly compare the manually and metallographically polished surfaces of CAD/CAM restorative materials for definitive restorations. Most studies have exclusively concentrated on either manual [8,39,41,42,43,44,45,46,47,48,49,50,51,52,53,54,55,56,57,58] or metallographic polishing techniques [37,38,59,60,61,62,63,64,65,66,67]. This methodological innovation allows an assessment of how effectively clinical polishing procedures achieve optimal surface outcomes [68].

The surface texture of restorative materials plays a critical role in clinically relevant characteristics, including the color stability, surface gloss, and degradation of the final restoration [69]. Additionally, the significant impact of both surface roughness and surface wettability on plaque accumulation—and, consequently, on periodontal inflammation and secondary caries—has been well documented [25,26,27,28]. Evaluating multiple 3D surface parameters, rather than relying solely on the amplitude parameters Ra and Sa, may provide a more comprehensive understanding of the characterization of the material’s unique surface topography [70,71,72]. Specifically, in the context of clinical dentistry, the Sdr, Sds, Sc, and Sv parameters may be related to a material’s potential for surface friction, gloss, fluid, and bacterial retention [35]. The significant effect of additional surface parameters is further emphasized by the fact that although Ra values above 0.2 μm are generally associated with increased bacterial attachment [10,51,73,74], exceptions to this trend have been reported [39,50,54,55].

All CAD/CAM restorative materials outperformed the direct composite resin in terms of surface roughness following manual polishing. Their industrial manufacturing process may partially explain this result; the polymerization of indirect restorative materials is carried out using high-heat and high-pressure techniques, or, in the case of additive manufacturing via vat polymerization. These methods result in more homogeneously polymerized structures with fewer irregularities [75,76,77,78]. Consequently, these more uniform surfaces may be associated with reduced discoloration, less plaque accumulation, and higher wear resistance, contributing to clinically desirable outcomes [25,26,27,28,69]. Contrarily, the incremental placement and intraoral light curing of direct resin composites may result in porosities and increased levels of unreacted monomers, compromising their clinical performance [79].

Shofu HC (SH) exhibited the most favorable surface roughness characteristics following manual polishing. This may be partially attributed to its filler content—reported at 61% by weight and 72% by volume [32,80]—and the hardness of its filler particles, silica powder and zirconium silicate, which are rated 7 to 7.5 on the Mohs hardness scale [81]. The coordinated abrasion of the relatively soft fillers and the surrounding organic matrix may result in a homogenous and smooth surface with fewer defects. Notably, no significant differences were observed between manual and metallographic polishing for SH across most roughness parameters, suggesting that the manufacturer’s recommended polishing protocol is effective in achieving a clinically sufficient surface finish. This is clinically significant, as it indicates predictable and reproducible outcomes without the need for extensive chairside adjustments.

The 3D-printed material VarseoSmile TriniQ (TQ) showed acceptable roughness values, particularly after metallographic polishing. Although limited data concerning its composition is available [82,83], the current results suggest that it can achieve a surface quality comparable to that of milled CAD/CAM materials, highlighting its potential as a restorative material for definitive restorations. However its high organic content and heterogeneous filler loading—validated by aromatic compounds and a complex inorganic peak at approximately 1100 cm^−1^—may influence its long-term performance. Therefore, further research is needed to verify its durability and clinical performance.

Vita Enamic (VE) features a three-dimensional ceramic scaffold that enables the interaction of filler particles. This bridging phenomenon, along with the irregularly shaped ceramic fillers with sizes up to 10 μm, have been scientifically documented [40,84]. Its inorganic filler content displays distinct hardness values, with aluminum oxide particles rated at 9 and silicon dioxide at 7 on the Mohs scale [81]. Additionally, VE exhibited the lowest relative intensity of organic phase peaks in the ATR-FTIR spectra. These microstructural features may explain the pronounced disparity in the decrease rates of Sa and Sz observed for VE after metallographic grinding. This finding suggests that although Vita Enamic can generally achieve a very smooth surface, as reflected by low Sa values, intermittently distributed surface irregularities—likely caused by isolated scratches on hard fillers or greater wear of the dispersed organic matrix—may persist. Under clinical conditions, such localized imperfections could contribute to plaque retention or surface degradation over time, despite a generally smooth surface profile. Finally, the significantly increased Sa, Sz, Sdr, Sc, and Sv roughness parameters of Vita Enamic compared with Shofu HC after manual polishing may be attributed to its inherent microstructural characteristics and the interaction with the specific polishers used.

Thus, surface roughness depends on both the material’s composition and the polishing system used [28,42,55,58,68,85,86,87,88]. The abrasive particles should exhibit hardness equal to or greater than that of the inorganic filler content of the resin-based materials [89]. Hardness, abrasive grain size, and polisher design influence the material’s surface microstructure [68,85,87,90]. Uniform polishing presents challenges for the dental practitioner due to disparities in the hardness of the organic matrix and the inorganic fillers, which compromise the homogeneous abrasion of both material phases [58,91]. The duration and applied pressure during polishing are additional influential factors affecting surface roughness [45,92]. This is further supported by our observation that manual polishing resulted in high standard deviations across all investigated surface parameters. Within each material, all groups—except SH—showed a significant roughness reduction after metallographic grinding. This suggests that the manufacturer-recommended polishing system for the milled, resin-based CAD/CAM material Shofu HC is efficient in achieving an initially optimal surface quality in clinical settings. For the remaining material groups, greater efforts should be made to develop clinically relevant polishing systems that effectively optimize surface texture.

Although direct comparisons to previously published data should be interpreted with caution due to differences in material types, polishing protocols, and surface roughness parameters, multiple studies support the findings of this in vitro study. Specifically, the increased roughness values of the hybrid ceramic material VE compared with milled CAD/CAM materials with dispersed fillers have been confirmed by several other studies [39,40,42,47]. However, divergent results are evident in the current literature. One study demonstrated inferior surface characteristics of Vita Enamic, even compared with two direct composite resins [39]. At the same time, another study found no statistically significant differences between milled CAD/CAM materials and a conventional composite resin [38]. Both observations contrast with the findings of our study. Additionally, one study exhibited no differences in surface roughness when comparing several milled CAD/CAM restorative materials [50]. One study reported a lower surface roughness for 3D-printed restoratives compared with hybrid ceramics [47], whereas another found no significant differences between milled and 3D-printed CAD/CAM materials [46]. Studies evaluating solely 3D-printed materials reported Ra values above the threshold of 0.2 μm [63,67]. A recent study reported baseline Ra values for VarseoSmile TriniQ up to 0.07 μm aligning with the findings of this study [48]. In this study, VarseoSmile TriniQ showed a comparable surface quality to SH and VE following optimal metallographic polishing, supporting its potential as a viable option for definitive restorations. Such discrepancies can be further interpreted through methodological factors, including specimen preparation techniques (e.g., milled versus low-speed precision cutting) and the surface measurement devices used (e.g., linear, contact versus 3D non-contact interferometric profilers, or atomic force microscopy). Additional factors contributing to the discrepancies include the polishing procedures applied (e.g., chairside polishing versus glazing, or metallographic grinding), the storage media (e.g., dry storage versus storage in distilled water), and 3D printing parameters such as layer thickness and model orientation.

While some concordance with prior data exists, the present study offers more detailed insights by employing a multi-parametric surface roughness analysis, which directly combines clinically relevant and laboratory polishing protocols. These elements reinforce the applicability of our findings to real-world restorative dentistry.

According to the literature, besides surface roughness, other factors such as surface topography, surface wettability, surface charge, surface chemical composition, and surface free energy demonstrate a decisive role in microbial cell attachment on dental materials [93,94,95,96]. It is well documented that bacteria tend to adhere more readily to surfaces that are slightly hydrophobic or hydrophilic [95].

Manual polishing resulted in higher contact angles compared with metallographic grinding for all materials, indicating increased surface irregularities that may trap air and reduce wettability—consistent with the Cassie–Baxter wetting model [97]. In contrast, surfaces polished under laboratory conditions likely follow the Wenzel regime, promoting better water spreading and reduced contact angles [98]. This phenomenon is within expectations, as it is related to the reduced micro-irregularities and the alteration of surface chemistry that occur following fine grinding.

Given that all manually polished surfaces exhibit contact angles below 90°, they can be characterized as moderately hydrophilic. In that context, the potential for bacterial attachment increases to varying degrees, depending on the material’s intrinsic characteristics [99]. Statistically significant differences in surface wettability among manually polished CAD/CAM materials reflect variations in surface chemistry and microstructure induced by manufacturer-recommended polishing protocols. Interestingly, this study revealed a significant inverse correlation between Sdr and contact angles, confirming that smoother and less complex surfaces enhance wettability. Under clinically relevant conditions, moderate hydrophilicity can promote the adsorption of salivary proteins, thereby facilitating microbial attachment. Concurrently, improving surface smoothness may not only enhance aesthetics, but also reduce plaque accumulation, thereby confining the risk of secondary caries.

There is scarce data on the water contact angles of resin-based CAD/CAM restorative materials in the literature. In alignment with the findings of this study, a recent investigation reported water contact angles of approximately 80° for all investigated CAD/CAM restoratives—whether subtractively or additively manufactured—after manual polishing using abrasive aluminum oxide-coated disks [55]. Wang et al. in 2025 investigated the surface wettability of several 3D-printed restorative materials and reported a wide range of water contact angles from 69.1° to 129.5°, attributing the variability to differences in material composition [67].

As with any in vitro study, several limitations should be acknowledged. Firstly, despite efforts to standardize the manual polishing procedure—using a single operator and new consumables per specimen—variations in force and motion have posed challenges. This reflects the real-time clinical conditions, but could have been controlled using a fixed polishing apparatus. Secondarily, although specimen fabrication was intended to replicate clinical workflows, the use of a low-speed precision cutting machine instead of milling units may have influenced surface topography. Additionally, surface roughness was evaluated immediately after polishing, but not after clinically relevant laboratory conditions, such as thermal cycling and wear simulation. In that manner, the long-term changes in surface properties remained unexplored. Furthermore, an SEM-EDS analysis could have provided a more robust characterization of the microstructure and filler distribution, particularly for the less-documented 3D-printed material. Finally, the complex conditions of the oral environment were not simulated. Physiological in vivo conditions could have altered surface energetics and bacterial interactions. Despite these limitations, the study design permits meaningful conclusions about the polishability and clinical performance of CAD/CAM restorative materials.

## 5. Conclusions

Within the limitations of this in vitro study, the following clinically relevant conclusions can be drawn: Subtractive and additive manufactured restorative materials demonstrated significantly lower surface roughness values compared with the conventional composite resin following manual polishing (e.g., manually polished FK with Sa: 163.45 nm; Sz: 1896.26 nm; and Sdr: 5.78 compared with manually polished VE with Sa: 88.72 nm; Sz: 1280.12 nm; and Sdr: 1.80 and manually polished TQ with Sa: 103.49 nm; Sz: 1043.51 nm; and Sdr: 1.68). Moreover, the type of material, polishing protocol, and their interaction significantly influence both surface texture and wettability.

Shofu HC exhibited the most consistent and favorable surface characteristics, even after manual polishing, indicating high polishability and clinical reliability (manually polished SH with Sa: 60.63 nm; Sz: 655.01 nm; and Sdr: 0.42). However, manufacturer-recommended polishing procedures for the rest of the materials may not fully replicate the surface quality achieved by optimal metallographic grinding, suggesting the need for developing advanced and material-specific polishing kits. In addition, all tested materials demonstrated moderate hydrophilicity, with surface roughness playing a key role in plaque accumulation. The 3D-printed materials show promising surface characteristics and may be suitable for definitive restorations (e.g., metallographically polished TQ with Sa: 48.86 nm; Sz: 754.08 nm; and Sdr: 0.74). Finally, in vivo studies are essential to validate these in vitro findings under real-time clinical conditions.

## Figures and Tables

**Table 1:** Materials used in the study.

Material	Abbreviation	Shade	Composition	Manufacturer
Filtek Z550 Direct composite restorative LOT: 11225283 [31]	FK	A2	Organic matrix: Bis-GMA, Bis-EMA, TEGDMA, PEGDMA, UDMA Inorganic fillers: 82 wt% inorganic fillers (non-agglomerated/non-aggregated 20 nm surface-modified silica particles, surface-modified zirconia/silica 0.1–10 μm)	3M ESPE, St. Paul, MN, USA
Shofu HC block CAD/CAM milled, Resin composite LOT: 111501 [32]	SH	A2 HT	Organic matrix: UDMA, TEGDMA Inorganic fillers: 61 wt% inorganic fillers (silica, zirconium silicate, and microfumed silica)	Shofu Inc., Kyoto, Japan
Vita Enamic CAD/CAM milled, hybrid ceramic LOT: 94630 [33]	VE	2M2 HT	Organic matrix: UDMA, TEGDMA Inorganic fillers: 86 wt% inorganic phase (primarily silicon dioxide and aluminum oxide and secondarily sodium, potassium, calcium oxide, boron trioxide and zirconia)	VITA Zahnfabrik, Bad Säckingen, Germany
VarseoSmile TriniQ CAD/CAM 3D-printed resin composite LOT: 601372 [34]	TQ	A2 Dentin	Organic matrix: Esterification products of 4,4′-isopropylidenediphenol, ethoxylated, and 2-methylprop-2-enoic acid: 55–80 wt%, benzeneacetic acid, alpha-oxo-, methyl ester < 5 wt%, diphenyl(2,4,6-trimethylbenzoyl) phosphine oxide < 2.5 wt% Inorganic fillers: ceramic fillers	Bego, Bremen, Germany

According to the manufacturers’ information. BisGMA: Bisphenol glycidyl dimethacrylate, BisEMA: Bishenol ethylene glycol diether dimethacrylate, TEGDMA: Triethyleneglycol dimethacrylate, PEGDMA: Polyethylene glycol dimethacrylate, UDMA: Urethane dimethacrylate.

**Table 2:** Materials used for the polishing procedures.

Polishing Systems	Composition	Manufacturer
Sof-Lex Finishing and Polishing System	Aluminum oxide abrasive particles (coarse, medium, fine, superfine)	3M ESPE, St. Paul, MN, USA
Super Snap	Aluminum oxide and silicon carbide particles serving as abrasive grains	Shofu Inc., Kyoto, Japan
DuraPolish	73% by weight aluminum oxide	Shofu Inc., Kyoto, Japan
DuraPolish DIA	67% diamond powder with ultrafine particle sizes smaller than 1 μm	Shofu Inc., Kyoto, Japan
VitaEnamic Polishing Set Clinical (two-step polishing system)	Silicon carbide abrasive particles for pre-polishing and diamond particles as abrasive grains for high-gloss polishing	VITA Zahnfabrik, Bad Säckingen, Germany
VitaPolish Hybrid	Diamond polishing paste Mixture of fatty acids, paraffin, and inorganic abrasive substances	VITA Zahnfabrik, Bad Säckingen, Germany
Opal L	High-luster polishing paste	Renfert GmbH, Hilzingen, Germany
Universal Polishing Paste	Water, aluminum oxide abrasives, solvent (hydrocarbons C10–C13), ammonium oleate, cocamide diethanolamine, ammonium hydroxide, pigments	Ivoclar Vivadent, Schaan, Lichtenstein
Silicon Carbide papers (800-, 1200-, 2400, 4000-grit)	Adhesive bonded silicon carbide grains	Struers, Copenhagen, Denmark
MD-NAP	Synthetic, short nap/diamond or oxide polishing, ≤1 μm grain size	Struers, Copenhagen, Denmark
DiaPro Nap R	Water-based, optimized with polycrystalline diamond solution/1 μm grain size	Struers, Copenhagen, Denmark

**Table 3:** Mean values and standard deviations (mean (SD)) of surface roughness parameters, along with post hoc comparisons among material groups and polishing procedures.

MATERIAL GROUP	Sa (nm)	Sz (nm)	Sdr (%)	Sds (1/mm^2^)	Sc (nm^3^/nm^2^)	Sv (nm^3^/nm^2^)
**MANUALLY POLISHED**						
**FK**	163.45 (13.12) a, A	1896.26 (270.60) a, A	5.78 (0.89) a, A	18,568.52 (1437.13) a, A	251.83 (40.11) a, A	22.92 (4.64) a, A
**SH**	60.63 (8.73) b, A	655.01 (103.37) b, A	0.42 (0.06) b, A	23,348.49 (2443.29) b, A	86.67 (15.72) b, A	10. 00(1.41) b, A
**VE**	88.72 (21.78) c, A	1280.12 (236.63) c, A	1.80 (0.92) c, A	22,521.08 (1182.05) b, A	110.83 (28.25) b, A	17.83 (3.66) c, A
**TQ**	103.49 (13.11) c, A	1043.51 (256.39) c, A	1.68 (0.64) c, A	21,325.56 (980.33) b, A	144.58 (19.16) c, A	17.17 (2.44) c, A
**METALLOGRAPHICALLY POLISHED**						
**FK**	57.19 (6.67) a, c, B	795.05 (78.18) a, B	0.91 (0.17) a, B	18,797.58 (1406.55) a, A	86.91 (10.86) a, B	8.83 (0.94) a, B
**SH**	59.69 (9.24) a, A	668.62 (107.13) b, A	0.46 (0.11) b, A	27,041.08 (2844.27) b, B	80.17 (15.02) a, c, A	10.80 (1.14) b, A
**VE**	35.37 (9.82) b, B	904.57 (262.69) a, B	0.72 (0.36) a, b, B	22,914.60 (4691.69) b, A	39.41 (11.97) b, B	8.86 (1.93) a, B
**TQ**	48.86 (10.30) c, B	754.08 (205.63) a, b, B	0.74 (0.32) a, b, B	25,507.03 (4202.46) b, B	65.66 (17.61) c, B	8.67 (0.98) a, B

Identical lowercase letters indicate mean values with no statistically significant differences among materials for each surface property within the same polishing condition, whereas identical uppercase letters indicate no significant differences between the two polishing conditions within the same material (*p* > 0.05).

**Table 4:** Mean values and standard deviations (mean (SD)) of contact angles, along with post hoc comparisons among material groups, after both manual and optimal metallographic polishing.

MATERIAL GROUP	Contact Angle (°) Manually Polished	Contact Angle (°) Metallographically Polished
**FK**	69.51 (1.87) a, A	52.65 (1.80) a, B
**SH**	75.75 (2.39) b, A	53.02 (1.19) a, B
**VE**	86.32 (1.23) c, A	47.16 (1.80) b, B
**TQ**	79.08 (1.96) d, A	66.26 (1.06) c, B

Identical lowercase letters indicate mean values with no statistically significant differences in surface wettability among materials within the same polishing condition, while identical uppercase letters indicate no significant differences within the same material between the two polishing conditions (*p* > 0.05).

## Data Availability

The raw data supporting the conclusions of this article will be made available by the authors on request.

## Supplementary Materials

The following supporting information can be downloaded at: https://www.mdpi.com/article/10.3390/ma18184222/s1. Table S1. Generalized Linear Model analysis for dependent variables Sa, Sz, Sdr, Sds, Sc, Sv, and contact angles.

## Abbreviations

The following abbreviations are used in this manuscript:

CAD/CAM	Computer-Aided Design/Computer-Aided Manufacturing
FK	Filtek Z550
VE	Vita Enamic
SH	Shofu HC
TQ	VarseoSmile TriniQ
PICN	Polymer-Infiltrated Ceramic Network
3D	Three-dimensional
SLA	Stereolithography
DLP	Digital Light Processing
LCD	Liquid Crystal Display
BisGMA	Bisphenol glycidyl dimethacrylate
BisEMA	Bishenol ethylene glycol diether dimethacrylate
TEGDMA	Triethyleneglycol dimethacrylate
PEGDMA	Polyethylene glycol dimethacrylate
UDMA	Urethane dimethacrylate
SiC	Silicon Carbide
RH	Relative humidity
ATR-FTIR	Attenuated Total Reflectance–Fourier Transformed Infrared Spectroscopy
GLM	Generalized linear model
SD	Standard Deviation
SEM-EDS	Scanning Electron Microscopy–Energy dispersive spectroscopy
Micro-CT	Micro-computed Tomography
