# Supplementary material for: Surface Characteristics of Subtractively and Additively Manufactured Restorative Materials for Definitive Restorations
Source: Materials (Basel). 2025 Sep 9;18(18):4222. doi: 10.3390/ma18184222 (PMC12471403; doi:10.3390/ma18184222)
Supplement: Supplementary file 1 [file materials-18-04222-s001.zip › materials-3848637-supplementary.pdf]

Table S1: Generalized Linear Model analysis for dependent variables Sa, Sz, Sdr, Sds, Sc, Sv and contact angles.

Dependent parameter	Predictors	*B* estimate	Wald χ^2	df	*p* - value	Interpretation
**Sa**<br>$\chi^2 = 234.37$, $df = 7$, $p < 0.001$<br><br>material (main effect): *Wald* $\chi^2 = 276.22$, $p < 0.001$<br><br>polishing procedure (main effect): *Wald* $\chi^2 = 494.00$, $p < 0.001$<br><br>material and polishing procedure (interaction): *Wald* $\chi^2 = 236.71$, $p < 0.001$	Intercept (manually polished FZ)	163.45	2280.35	1	$p < 0.001$	SH, VE and TQ show a significant decrease in Sa relative to the reference group<br><br>Metallographic polishing resulted in a significant decrease in Sa compared to manual polishing<br><br>The effect of polishing on Sa depended on the material type<br><br>The reduction effect from metallographic polishing was mitigated depending on the material type, with a greater moderation presented for SH
	SH	– 102.82	451.17	1	$p < 0.001$	
	VE	–74.73	238.36	1	$p < 0.001$	
	TQ	–59.96	153.42	1	$p < 0.001$	
	metallographic polishing	–106.26	481.85	1	$p < 0.001$	
	metallographically polished SH	+105.31	236.67	1	$p < 0.001$	
	metallographically polished VE	+52.91	59.75	1	$p < 0.001$	
	metallographically polished TQ	+51.62	56.86	1	$p < 0.001$	
**Sz**<br>$\chi^2 = 154.35$, $df = 7$, $p < 0.001$<br><br>material (main effect): *Wald* $\chi^2 = 158.17$, $p < 0.001$<br><br>polishing procedure (main effect): *Wald* $\chi^2 = 120.20$, $p < 0.001$<br><br>material and polishing procedure (interaction): *Wald* $\chi^2 = 104.84$, $p < 0.001$	Intercept (manually polished FZ)	1896.26	1125.72	1	$p < 0.001$	SH, VE and TQ show a significant decrease in Sz relative to the reference group<br><br>Metallographic polishing resulted in a significant decrease in Sz compared to manual polishing<br><br>The effect of polishing on Sz depended on the material type<br><br>The reduction effect from metallographic polishing was mitigated depending on the material type, with a greater moderation presented for SH
	SH	-1241.25	241.17	1	$p < 0.001$	
	VE	-616.14	59.42	1	$p < 0.001$	
	TQ	-852.75	113.83	1	$p < 0.001$	
	metallographic polishing	-1101.21	189.82	1	$p < 0.001$	
	metallographically polished SH	+1114.82	97.27	1	$p < 0.001$	
	metallographically polished VE	+725.65	41.21	1	$p < 0.001$	
	metallographically polished TQ	+811.77	51.56	1	$p < 0.001$	
**Sdr**<br>$\chi^2 = 233.65$, $df = 7$, $p < 0.001$<br><br>material (main effect):	Intercept (manually polished FZ)	5.79	1504.22	1	$p < 0.001$	SH, VE and TQ show a significant decrease in Sdr relative to the reference group<br><br>Metallographic polishing resulted in a significant decrease in Sdr
	SH	-5.37	648.09	1	$p < 0.001$	
	VE	-3.98	356.63	1	$p < 0.001$	

Wald $\chi^2 = 419.05$, $p < 0.001$	TQ	-4.10	378.30	1	$p < 0.001$	compared to manual polishing
polishing procedure (main effect): *Wald* χ^2 = 262.81, $p < 0.001$	metallographic polishing	-4.87	533.62	1	$p < 0.001$	The effect of polishing on Sdr depended on the material type
	metallographically polished SH	+4.93	272.44	1	$p < 0.001$	The reduction effect from metallographic polishing was mitigated depending on the material type, with a greater moderation presented for SH
material and polishing procedure (interaction): *Wald* $\chi^2 = 316.84$, $p < 0.001$	metallographically polished VE	+3.79	161.55	1	$p < 0.001$	
	metallographically polished TQ	+3.94	174.10	1	$p < 0.001$	
**Sds**	Intercept (manually polished FZ)	18568.53	600.47	1	$p < 0.001$	SH, VE and TQ show a significant increase in Sds relative to the reference group
$\chi^2 = 71.61$, $df = 7$, $p < 0.001$	SH	+4779.69	19.90	1	$p < 0.001$	
material (main effect): *Wald* $\chi^2 = 79.13$, $p < 0.001$	VE	+3952.56	13.60	1	$p < 0.01$	No statistically significant increase in Sds for metallographic polishing compared to manual polishing
polishing procedure (main effect): *Wald* χ^2 = 15.72, $p < 0.001$	TQ	+2757.03	6.62	1	$p < 0.001$	
	metallographic polishing	+229.06	0.05	1	$p > 0.05$	
material and polishing procedure (interaction): *Wald* $\chi^2 = 11.56$, $p < 0.01$	metallographically polished SH	+3463.54	5.22	1	$p < 0.05$	The effect of polishing on Sds depended on the material type for SH and TQ, but not for VE
	metallographically polished VE	+164.46	0.01	1	$p > 0.05$	
	metallographically polished TQ	+3952.42	6.80	1	$p < 0.01$	
**Sc**	Intercept (manually polished FZ)	251.83	1739.39	1	$p < 0.001$	SH, VE and TQ show a significant decrease in Sc relative to the reference group
$\chi^2 = 217.42$, $df = 7$, $p < 0.001$	SH	-165.17	374.10	1	$p < 0.001$	
material (main effect): *Wald* $\chi^2 = 299.50$, $p < 0.001$	VE	-141.00	272.63	1	$p < 0.001$	Metallographic polishing resulted in a significant decrease in Sc compared to manual polishing
	TQ	-107.25	157.74	1	$p < 0.001$	
polishing procedure (main effect): *Wald* χ^2 = 354.91, $p < 0.001$	metallographic polishing	-164.92	372.67	1	$p < 0.001$	The effect of polishing on Sc depended on the material type
	metallographically polished SH	+158.42	172.07	1	$p < 0.001$	The reduction effect from metallographic polishing was mitigated depending on the material type, with a greater moderation presented for SH
material and polishing procedure (interaction): *Wald* $\chi^2 = 173.98$, $p < 0.01$	metallographically polished VE	+93.50	59.94	1	$p < 0.001$	
	metallographically polished TQ	+86.00	50.71	1	$p < 0.001$	
**Sv**	Intercept (manually polished FZ)	22.92	1104.36	1	$p < 0.001$	SH, VE and TQ show a significant decrease in Sv relative to the reference group
$\chi^2 = 163.74$, $df = 7$, $p < 0.001$	SH	-12.92	175.42	1	$p < 0.001$	

material (main effect): *Wald* $\chi^2 = 62.47$, $p < 0.001$	VE	-5.08	27.17	1	$p < 0.001$	Metallographic polishing resulted in a significant decrease in Sv compared to manual polishing
	TQ	-5.75	34.76	1	$p < 0.001$	
polishing procedure (main effect): *Wald* $\chi^2 = 247.34$, $p < 0.001$	metallographic polishing	-14.08	208.54	1	$p < 0.001$	The effect of polishing on Sv depended on the material type
	metallographically polished SH	+14.97	117.76	1	$p < 0.001$	
material and polishing procedure (interaction): *Wald* $\chi^2 = 122.68$, $p < 0.01$	metallographically polished VE	+5.11	13.72	1	$p < 0.001$	The reduction effect from metallographic polishing was mitigated depending on the material type, with a greater moderation presented for SH
	metallographically polished TQ	+5.58	16.39	1	$p < 0.001$	
**Water contact angles**	Intercept (manually polished FZ)	69.51	21428.78	1	$p < 0.001$	SH, VE and TQ show a significant increase in contact angles relative to the reference group
$\chi^2 = 401.25$, $df = 7$, $p < 0.001$	SH	+6.24	86.42	1	$p < 0.001$	Metallographic polishing resulted in a significant decrease in contact angles compared to manual polishing
material (main effect): *Wald* $\chi^2 = 635.56$, $p < 0.001$	VE	+16.82	627.00	1	$p < 0.001$	
	TQ	+9.57	203.06	1	$p < 0.001$	
polishing procedure (main effect): *Wald* $\chi^2 = 4648.74$, $p < 0.001$	metallographic polishing	-16.86	630.05	1	$p < 0.001$	The effect of polishing on contact angles depended on the material type
	metallographically polished SH	-5.88	38.35	1	$p < 0.001$	
material and polishing procedure (interaction): *Wald* $\chi^2 = 892.91$, $p < 0.01$	metallographically polished VE	-22.31	551.67	1	$p < 0.001$	
	metallographically polished TQ	+4.04	18.08	1	$p < 0.001$	
